# Intraoperative hypotension in non-emergency decompression surgery for cervical spondylosis: The role of chronic arterial hypertension

**DOI:** 10.3389/fmed.2022.943596

**Published:** 2022-10-18

**Authors:** Ting-Yun Chiang, Yen-Kai Wang, Wen-Cheng Huang, Shiang-Suo Huang, Ya-Chun Chu

**Affiliations:** ^1^Department of Anesthesiology, Taipei Veterans General Hospital, Taipei City, Taiwan; ^2^School of Medicine, National Yang Ming Chiao Tung University, Hsinchu, Taiwan; ^3^Department of Neurosurgery, Neurological Institute, Taipei Veterans General Hospital, Taipei City, Taiwan; ^4^Department of Pharmacology, Institute of Medicine, Chung Shan Medical University, Taichung, Taiwan; ^5^Department of Pharmacy, Chung Shan Medical University Hospital, Taichung, Taiwan

**Keywords:** cervical spondylosis, chronic hypertension, intraoperative hypotension, myelopathy, non-emergency, ossification of posterior longitudinal ligament

## Abstract

**Background:**

Cervical spondylotic myelopathy and chronic hypertension show a cause-effect relationship. Hypertension increases cardiovascular risk and is associated with intraoperative hypotension. We aimed to evaluate intraoperative hypotension in patients undergoing non-emergency decompression surgery for cervical spondylosis and its association with clinical myelopathy and chronic arterial hypertension.

**Methods:**

This retrospective cohort study used healthcare data of adult patients undergoing cervical spine surgeries at Taipei Veterans General Hospital from 2015 to 2019. The primary outcomes were the incidence of intraoperative hypotension and predictive factors, and the secondary outcomes were the association of intraoperative hypotension and postoperative adverse outcomes in the surgical population.

**Results:**

Among the 1833 patients analyzed, 795 (43.4%) required vasopressor treatment and 342 (18.7%) showed persistent hypotension. Factors independent associated with hypotension after anesthetic induction were age [odds ratio (OR), 1.15; 95% confidence interval (CI), 1.07-1.23 per 5 years, *P* < 0.001], male sex (OR, 1.63; 95% CI, 1.21-2.19, *P* < 0.001), chronic hypertension (OR, 1.77; 95% CI, 1.32-2.38, *P* < 0.001), upper cervical spine level C0-2 treated (OR, 3.04; 95% CI, 1.92-4.84, *P* < 0.001 vs. C3-T1), and increased number of spine segments treated (OR, 1.43; 95% CI 1.26-1.63, *P* < 0.001). Patients who developed intraoperative hypotension experienced more acute postoperative kidney injury (OR, 7.90; 95% CI, 2.34–26.63, *P* < 0.001), greater need for intensive care (OR, 1.80; 95% CI, 1.24–2.60, *P* = 0.002), and longer admission after surgery (1.09 days longer, 95% CI 0.06-2.12, *P* = 0.038).

**Conclusion:**

Intraoperative hypotension is common even in non-emergency cervical spine surgery. A history of hypertension independently predicted intraoperative hypotension. Prompt assessments for identifiable features can help ameliorate intraoperative hypotension.

## Introduction

Intraoperative hypotension is associated with negative postoperative outcomes, and even episodes of as little as 1–5 min can show unfavorable effects (1, 2). Although intraoperative hypotension may be caused by patient dehydration, anesthetics, and surgical factors (3), an understanding of the risk factors can facilitate early detection and timely treatment to minimize the hypotensive period. Factors associated with post-induction hypotension include a low pre-induction systolic arterial pressure (SAP), age, emergency surgery, male sex, high American Society of Anesthesiologists physical status (ASA-PS) class, anesthetic dosage for induction, and duration of surgery (4–6). Chronic arterial hypertension has been recently reported to predict post-induction hypotension or intraoperative hypotension (7, 8). Dysfunction of the autonomic nervous system has been assumed to be one of the mechanisms underlying impaired blood pressure regulation and cardiovascular events (9), including the risk of hemodynamic instability after anesthetic induction (10). A preoperative high sympathetic tone may mask preload insufficiency, leading to hypotension due to vasoplegia by the suppressed sympathetic tone after induction (10). Furthermore, autonomic dysfunction also leads to inadequate response to tissue perfusion, resulting in the persistent hypotension (10). Recognition of patient comorbidities related to autonomic nerve dysfunction can optimize prevention and shorten the hypotensive period.

Cervical spondylosis results in gradual cord compression, poor vascular perfusion, and ultimately, cervical myelopathy. Surgical decompression is currently the treatment of choice for cord compression. Surgical decompression has been reported to not only improve neurological disability but also treat concomitant hypertension (11–14); chronic hypertensive patients become normotensive or show a reduction in the severity of hypertension after surgery (11–14). Persistent hypertension has been reported to be associated with the severity of clinical manifestation and imaging evidence of myelopathy (15). Cervical spondylosis has also been reported to be associated with a higher risk of arrhythmia (16), acute coronary syndrome (17), and migraine (18), which were considered to result from autonomic dysfunction (14). Patients with cervical myelopathy experienced definite autonomic nervous dysfunction in comparison with healthy age- and sex-matched controls (19), and surgical decompression improved autonomic regulation (13, 14). A cause-effect relationship between myelopathy and hypertension was proposed, but the pathognomonic mechanism has not been fully established. The anatomic distribution of sympathetic nerve fibers in the cervical dura mater and posterior longitudinal ligament (PLL) provides a pathological basis for autonomic neuropathy (20), which can be assumed to be attributable to the sustained sympathetic stimulation induced by chronic irritation by the spurs or herniated disk in patients with cervical cord compression with autonomic symptoms (21, 22).

Previous predictions of intraoperative hypotension did not focus on a specific surgical population. Based on the proposed cause-effect relationship between cervical spondylotic myelopathy and chronic hypertension and the possible dysfunction of the autonomic nervous system (13), we hypothesized that patients with chronic hypertension are more susceptible to intraoperative hypotension. Thus, we conducted a retrospective cohort study to characterize intraoperative hypotension in the patient population and the associated risk factors pertaining to surgical features.

## Materials and methods

### Ethics approval

The protocol of this retrospective cohort study was reviewed and approved by the institutional review board of Taipei Veterans General Hospital (ethics committee number 2018-08-001B, date of approval: 08/08/2018). Owing to the retrospective nature of this study and anonymization of data, the need for informed consent was waived.

We extracted and analyzed data from our digitized medical and anesthesia records, which included biometric, medical, procedural, and physiological variables of patients undergoing cervical spine surgery and general anesthesia at our hospital (Taipei Veterans General Hospital, Taipei, Taiwan) between January 1, 2016, and December 31, 2019.

### Patient population

An unselected sample of 2,466 adult patients, who underwent general anesthesia to facilitate non-emergency cervical spine surgeries, were enrolled during the study period. We excluded patients with a diagnosis related to trauma history, diagnosis unrelated to cervical spondylosis, emergency surgery, vasopressor treatment preoperatively, or admission to an intensive care unit due to hemodynamic instability before surgery. Data from repeated surgeries were also excluded, and only the data from the first surgery for these patients were included in the data analysis. Anesthesia records were also reviewed to exclude cases of suspected anaphylaxis in the presence of skin manifestations such as urticaria or rash.

### Definition of intraoperative arterial hypotension

Intraoperative hypotension occurred frequently, although most cases (73.4%) involved minor single events (6). We aimed to address the potentially severe and/or persisting hypotensive state (6). Thus, hypotension was defined as at least two readings of SAP < 80 mmHg and more than two incidents (≥3) of bolus administration or vasopressor infusion (23, 24). The entity of hypotension was distinguished according to the time period during which it occurred: (i) from induction of general anesthesia until surgical incision, termed post-induction hypotension; and (ii) from surgical incision until the end of the surgery, termed post-incision hypotension.

### Data acquisition

The available digitized anesthesia records were automatically scrutinized for patients who met the inclusion criteria, and only the data of eligible patients were included in subsequent analyses. Missing data were minimized by manual re-examination of the dataset and re-collection from medical records. Basic medical status, including age, sex, body mass index, ASA-PS classification, relevant trauma history, emergency status, pre-existing comorbidities, and long-term medication, especially anti-hypertensive medications, were extracted. Additional data, including perioperative surgery- or anesthesia-related information and hemodynamic measurements, were manually transferred into evaluable data for this study. The records were examined for the doses and frequencies of administered anesthetics and cardiovascular drugs (e.g., catecholamines), and arterial blood pressure (ABP) readings were distinguished separately for the post-induction and post-incision periods. For non-invasive intermittent ABP measurements, we used an oscillometric device incorporated in the monitoring system Infinity Delta (Drager Medical GmbH, Lubeck, Germany) or Carescape Monitor B850 (GE Healthcare, Helsinki, Finland) set to a standardized 5-min measurement interval or at the discretion of the anesthetists. Continuous invasive ABP measurements were obtained using arterial catheters and pressure transducers. Pre-induction ABP was documented as the measurement before the administration of anesthetics for induction. Intraoperative ABP and frequency of vasopressor administration were counted every time the anesthetist recorded a bolus and separately counted for the two time periods. The doses of the induction anesthetics were restricted to propofol and normalized to body weight. Vasopressors were administered at the discretion of the attending anesthetists and expressed as the sum of the administered boluses and total amounts during each observation period.

Postoperative in-hospital complications, need for intensive care, ICU stay period, postoperative admission period, and in-hospital mortality rates were compared between patients with and without intraoperative hypotension. In-hospital complications included (i) acute kidney injury, defined as per the KDIGO guidelines (25); (ii) respiratory complications such as glottic edema-related stridor/dyspnea, pneumonia, respiratory failure or acute respiratory distress syndrome, and pleural effusion; (iii) cardiovascular complications such as myocardial ischemia or infarction, arrhythmia, shock, cerebrovascular accident/stroke, heart failure, and pulmonary edema; and (iv) surgical complications such as the formation of hematomas, dura tear, esophageal injuries, cervical sympathetic chain injuries, and screw malpositioning.

### Outcomes

The primary outcomes were the incidence of persistent intraoperative hypotension and the feature factors associated with hypotension. Feature factors included chronic arterial hypertension, clinical myelopathy, cervical spine level (upper vs. lower cervical spine) treated, number of segments treated, and ossification of PLL (OPLL). The secondary outcomes were the association of intraoperative hypotension and postoperative adverse outcomes in the surgical population. Clinical myelopathy was defined based on a diagnosis by the spine surgeon, or the presence of difficulty with fine motor skills, unsteady gait or loss of ability to move quickly, or urinary or bowel function recorded in medical records.

### Statistical analysis

Categorical baseline characteristics of patients were compared between groups (e.g., hypotension vs. non-hypotension) by using Fisher’s exact test. Continuous baseline patient characteristics are presented as mean [standard deviation (SD)] or median [interquartile range (IQR)]. The mean and median data were compared between the groups by using the independent-sample *t*-test and Mann–Whitney *U*-test, respectively. A series of univariate logistic regression analyses were conducted to evaluate the potential relationship between the feature characteristics and the risk of post-induction/post-incision hypotension. Variables with a significance level of less than 0.20 were introduced into a multivariable logistic regression model with backward elimination. The variables retained in the final step of the multivariable model were deemed to be independently associated with post-induction/post-incision hypotension.

Finally, the association between intraoperative hypotension and postoperative outcomes was investigated using logistic regression analysis for categorical outcomes (e.g., acute kidney injury) or linear regression analysis for continuous outcomes (e.g., post-operation days). Several clinically relevant factors were adjusted in the multivariate analysis, including age, sex, body mass index, ASA-PS, number of spine segments treated, blood loss, hypertension, and diabetes. A 2-sided *P*-value of < 0.05 was considered statistically significant. Statistical analyses were conducted using SPSS 26 software for statistical analysis (IBM SPSS, Chicago, IL, USA).

## Results

**FIGURE 1:**
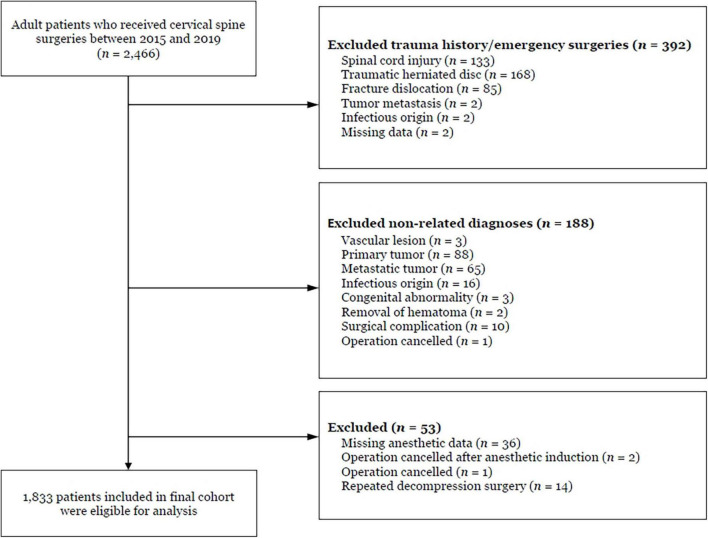
Flow chart of patient enrollment.

Patient selection is shown in the flowchart (Figure 1). A total of 1,833 patients were included in the final analysis, of which 795 (43.4%) required vasopressor treatment and 342 (18.7%) met the criteria for persistent hypotension. Of these, 254 (13.9%) experienced persistent post-induction hypotension, 277 (15.1%) experienced post-incision hypotension, and 189 (10.3%) experienced hypotension during both periods (Table 1).

**TABLE 1:** Demographic and clinical characteristics stratified by the presence of intraoperative hypotension.

Variable	Total (*n* = 1,833)	With hypotension (*n* = 342)	Without hypotension (*n* = 1,491)	*P*
Age, years, median (IQR)	61 (52–68)	65 (58–71)	59 (51–67)	< 0.001
Male, *n* (%)	1,052 (57.4)	231 (67.5)	821 (55.1)	< 0.001
Body mass index, kg/m^2^, median (IQR)	25 (22.9–27.8)	26 (23.4–28.1)	25 (22.8–27.7)	0.030
ASA, *n* (%)				< 0.001
1&2	1,480 (80.7)	243 (71.1)	1,237 (83.0)	
3&4	353 (19.3)	99 (28.9)	254 (17.0)	
Myelopathy, *n* (%)	754 (41.1)	159 (46.5)	595 (39.9)	0.026
Cervical level treated, *n* (%)				< 0.001
C0-C2	108 (5.9)	41 (12.0)	67 (4.5)	
C3-T1	1,725 (94.1)	301 (88.0)	1,424 (95.5)	
Number of level treated, median (IQR)	2 (2–3)	3 (2–4)	2 (2–3)	< 0.001
Current smoker, *n* (%)	419 (22.9)	80 (23.4)	339 (22.7)	0.795
Pre-induction SBP, mmHg, median (IQR)	143 (130–157)	146 (134–159)	143 (129–157)	0.007
Propofol during anesthetic induction, mg/kg, mean (SD)	1.99 (0.48)	1.89 (0.45)	2.01 (0.48)	< 0.001
Blood loss, ml, median (IQR)	100 (50–150)	100 (50–300)	50 (50–150)	< 0.001
**Comorbidity, *n* (%)**				
Hypertension	787 (42.9)	204 (59.6)	583 (39.1)	< 0.001
OPLL	232 (12.7)	63 (18.4)	169 (11.3)	< 0.001
Diabetes	360 (19.6)	84 (24.6)	276 (18.5)	0.011
Chronic kidney disease	42 (2.3)	11 (3.2)	31 (2.1)	0.205
Congestive heart failure	10 (0.5)	4 (1.2)	6 (0.4)	0.082
Coronary heart disease	166 (9.1)	39 (11.4)	127 (8.5)	0.094
**Medication, *n* (%)**				
Vasodilator	59 (3.2)	15 (4.4)	44 (3.0)	0.175
CCB	392 (21.4)	86 (25.1)	306 (20.5)	0.060
ACEi/ARB	411 (22.4)	82 (24.0)	329 (22.1)	0.445
Diuretic/Thiazide	83 (4.5)	19 (5.6)	64 (4.3)	0.311
Beta blocker	209 (11.4)	33 (9.6)	176 (11.8)	0.258
Alpha blocker	34 (1.9)	6 (1.8)	28 (1.9)	0.879
**Hypotension and treatments**				
Lowest SBP, mmHg, median (IQR)	88 (72–98)	72 (69–75)	90 (75–98)	< 0.001
Lowest MBP, mmHg, median (IQR)	65 (55–72)	54 (50–57)	68 (57–74)	< 0.001
Duration of hypotension (SBP < 80), min, median (IQR)	0 (0–8)	15 (10–20)	0 (0–5)	< 0.001
Vasopressor treatment, *n* (%)				< 0.001
None	1,038 (56.6)	0 (0.0)	1,038 (69.6)	
Boluses administration	755 (41.2)	302 (88.3)	453 (30.4)	
Infusion with boluses	40 (2.2)	40 (11.7)	0 (0.0)	
No. of boluses of vasopressor, median (IQR)	0 (0–2)	3 (2–4)	0 (0–1)	< 0.001
Total doses of bolus ephedrine, mg, mean (SD)	6.1 (9.6)	20.9 (11.3)	2.7 (4.7)	< 0.001
Total doses of bolus norepinephrine, μg, mean (SD)	0.5 (5.3)	2.9 (12.1)	0 (0.1)	< 0.001

SD, standard deviation; IQR, interquartile range; ASA, American Society of Anesthesiologists; SBP, systolic blood pressure; OPLL, ossification of the posterior longitudinal ligament; CCB, calcium-channel blocker; ACEi, angiotensin-converting enzyme inhibitor; ARB, angiotensin receptor blocker. Data are presented as frequency (percentage) or mean (standard deviation) and median (25th percentile–75th percentile).

### Population characteristics

The median age of all patients was 61 years (IQR, 52–68 years), with a male predominance in sex distribution (57.4%). The distribution of ASA-PS classes I-II and III-IV was 80.7 and 19.3%, respectively. Among the patients, 41% (*n* = 754) were clinically diagnosed with myelopathy. The treated cervical vertebrae were predominantly in the lower cervical levels [Cervical vertebra (C)3- thoracic vertebra (T)1; 94.1%] rather than the upper cervical levels (C0-2; 5.9%). The median number of spine segments treated was 2 (IQR, 2–3). The most prevalent comorbidity was chronic arterial hypertension (42.9%). The median pre-induction SBP was 143 mmHg (IQR, 130–157 mmHg) (Table 1). In addition, 1,448 patients (79%) underwent arterial catheterization for invasive continuous ABP measurements.

In comparison with patients without intraoperative hypotension, those with intraoperative hypotension had the following characteristics: older, greater male predominance, a higher ASA-PS class, comorbidity such as myelopathy, ossification of the posterior longitudinal ligament (OPLL), diabetes and chronic arterial hypertension (59.6 vs. 39.1% in patients without intraoperative hypotension), treated at upper cervical spine levels (C0-2), a higher pre-induction SBP, and a greater blood loss. Patients with hypotension are more likely to receive vasopressor infusion (11.7 vs. 0%), boluses of vasopressor [3(2–4) vs. 0(0–1)], experience a longer duration of SBP < 80 mmHg [15 (10–20) vs. 0 (0–5) min], and had a lower lowest blood pressure measurement (Table 1).

### Factors associated with post-induction and post-incision hypotension

Demographic comparisons between patients with and without hypotension during the post-induction and post-incision periods are listed in Table 2. In comparison with patients without hypotension, those with post-induction hypotension were older, showed greater male predominance, had higher ASA-PS, were more likely to have clinical myelopathy, were more often treated with operations of upper cervical spine levels and increased number of segments, showed higher prevalence of chronic arterial hypertension (61.4 vs. 40.0%), OPLL, diabetes, and congestive heart failure, received fewer amounts of propofol during induction, and were less likely to receive beta-blockers. The results of the univariate logistic regression analyses for the crude relationship between the baseline characteristics and risks of post-induction hypotension and post-incision hypotension are provided in Supplementary Table 1.

**TABLE 2:** Patient demographics in the post-induction and post-incision hypotension groups.

	Post-induction hypotension			Post-incision hypotension		
Variable	Presence (*n* = 254)	Absence (*n* = 1,579)	*P*	Presence (*n* = 277)	Absence (*n* = 1,556)	*P*
Age, years, median (IQR)	65 (58–71)	60 (51–68)	< 0.001	65 (59–71)	59 (51–68)	< 0.001
Male, *n* (%)	172 (67.7)	880 (55.7)	< 0.001	189 (68.2)	863 (55.5)	< 0.001
Body mass index, kg/m^2^, median (IQR)	25.8 (23.5–28.1)	25.1 (22.8–27.7)	0.037	25.9 (23.3–28.3)	25.1 (22.9–27.7)	0.035
ASA, *n* (%)			< 0.001			< 0.001
1&2	184 (72.4)	1,296 (82.1)		194 (70.0)	1,286 (82.6)	
3&4	70 (27.6)	283 (17.9)		83 (30.0)	270 (17.4)	
Clinical myelopathy, *n* (%)	119 (46.9)	635 (40.2)	0.046	130 (46.9)	624 (40.1)	0.033
Cervical level treated, *n* (%)			< 0.001			< 0.001
C0-C2	37 (14.6)	71 (4.5)		35 (12.6)	73 (4.7)	
C3-T1	217 (85.4)	1,508 (95.5)		242 (87.4)	1,483 (95.3)	
No. of spine level treated, median (IQR)	3 (2–4)	2 (2–3)	< 0.001	3 (2–4)	2 (2–3)	< 0.001
Current smoker, *n* (%)	61 (24.0)	358 (22.7)	0.636	64 (23.1)	355 (22.8)	0.916
Pre-induction SBP, mm-Hg, median (IQR)	145 (134–157)	143 (129–157)	0.108	145 (134–157)	143 (129–157)	0.040
Propofol/Kg, mg, mean (SD)	1.9 (0.5)	2.0 (0.5)	< 0.001	–	–	–
Blood loss, ml, median (IQR)	–	–	–	100 (50–300)	50 (50–150)	< 0.001
**Comorbidity, *n* (%)**						
Hypertension	156 (61.4)	631 (40.0)	< 0.001	170 (61.4)	617 (39.7)	< 0.001
OPLL	49 (19.3)	183 (11.6)	< 0.001	56 (20.2)	176 (11.3)	< 0.001
Diabetes	62 (24.4)	298 (18.9)	0.039	68 (24.5)	292 (18.8)	0.026
Chronic kidney disease	10 (3.9)	32 (2.0)	0.059	10 (3.6)	32 (2.1)	0.111
Congestive heart failure	4 (1.6)	6 (0.4)	0.016	2 (0.7)	8 (0.5)	0.665
Coronary heart disease	28 (11.0)	138 (8.7)	0.239	33 (11.9)	133 (8.5)	0.072
**Medication, *n* (%)**						
Vasodilator	9 (3.5)	50 (3.2)	0.752	14 (5.1)	45 (2.9)	0.060
CCB	59 (23.2)	333 (21.1)	0.440	66 (23.8)	326 (21.0)	0.282
ACEi/ARB	58 (22.8)	353 (22.4)	0.865	65 (23.5)	346 (22.2)	0.651
Diuretic/Thiazide	16 (6.3)	67 (4.2)	0.144	14 (5.1)	69 (4.4)	0.648
Beta blocker	19 (7.5)	190 (12.0)	0.034	28 (10.1)	181 (11.6)	0.462
Alpha blocker	5 (2.0)	29 (1.8)	0.885	4 (1.4)	30 (1.9)	0.582
**Hypotension and treatments**						
Lowest SBP, mmHg, median (IQR)	72 (68–75)	89 (75–98)	< 001	72 (68–75)	90 (75–98)	< 0.001
Lowest MBP, mmHg, median (IQR)	54 (50–56)	67 (56–74)	< 0.001	54 (50–57)	68 (57–74)	< 0.001
Duration of hypotension (SBP < 80), min, median (IQR)	18 (15–25)	0 (0–5)	< 001	15 (10–25)	0 (0–5)	< 0.001
Vasopressor treatment, *n* (%)			< 0.001			< 0.001
None	0 (0.0)	1,038 (65.7)		0.0 (0.0)	1,038 (66.7)	
Boluses administration	214 (84.3)	541 (34.3)		241 (87.0)	514 (33.0)	
Infusion with boluses	40 (15.7)	0 (0.0)		36 (13.0)	4 (0.3)	
No. of boluses of vasopressor, median (IQR)	3.0 (3–5)	0 (0–1)	< 0.001	3 (2–4)	0 (0–1)	< 0.001
Total doses of bolus ephedrine, mg, mean (SD)	24.2 (10.6)	3.2 (5.2)	< 0.001	21.2 (12.1)	3.4 (5.8)	< 0.001
Total doses of bolus norepinephrine, μg, mean (SD)	3.7 (13.9)	0.03 (0.63)	< 0.001	3.4 (13.3)	0.03 (0.69)	< 0.001

MD, standard deviation; IQR, interquartile range; ASA, American Society of Anesthesiologists; SBP, systolic blood pressure; OPLL, ossification of the posterior longitudinal ligament; CCB, calcium-channel blocker; ACEi, angiotensin-converting enzyme inhibitor; ARB, angiotensin receptor blocker; Data are presented as frequency (percentage) or mean ± standard deviation and median (25th percentile–75th percentile).

**FIGURE 2:**
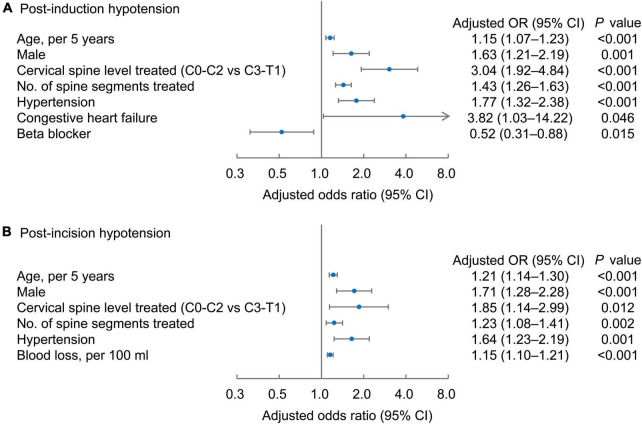
Risk factors independently associated with post-induction **(A)** and post-incision hypotension **(B)**. Forest plots of the adjusted odd ratios (dots) with 95% confidence intervals (95% CI; whiskers) of the multivariable regression models, with post-induction or post-incision hypotension as the dependent variable.

Factors independently associated with an increased risk for post-induction hypotension were as follows: Older age [odds ratio (OR), 1.15; 95% confidence interval (CI), 1.07–1.23 per 5 years, *P* < 0.001], male sex (OR, 1.63; 95% CI, 1.21–2.19, *P* = 0.001), upper cervical spine level (C0-2) treated (OR, 3.04; 95% CI, 1.92–4.84, *P* < 0.001 vs. level C3-T1), increased number of spine segments treated (OR, 1.43; 95% CI, 1.26–1.63, *P* < 0.001), history of chronic hypertension (OR, 1.77; 95% CI, 1.32–2.38, *P* < 0.001), and of congestive heart failure (OR, 3.82; 95% CI, 1.03–14.22, *P* = 0.046). Beta- blocker use was associated with a reduced risk of post-induction hypotension (OR, 0.52; 95% CI 0.31–0.88, *P* = 0.015) (Figure 2A). Factors associated with an increased risk of post-incision hypotension were as follows: older age (OR, 1.21; 95% CI, 1.14–1.30 per 5 years, *P* < 0.001), male sex (OR, 1.71; 95% CI, 1.28–2.28, *P* < 0.001), upper cervical spine levels (C0-2) treated (OR, 1.85; 95% CI, 1.142.99, *P* = 0.012), increased number of spine segments treated (OR, 1.23; 95% CI, 1.08–1.41, *P* = 0.002), greater blood loss (OR, 1.15; 95% CI, 1.10–1.21, *P* < 0.001), and the history of hypertension (OR, 1.64; 95% CI, 1.23–2.19, *P* = 0.001) (Figure 2B).

#### Postoperative outcomes by intraoperative hypotension

**FIGURE 3:**
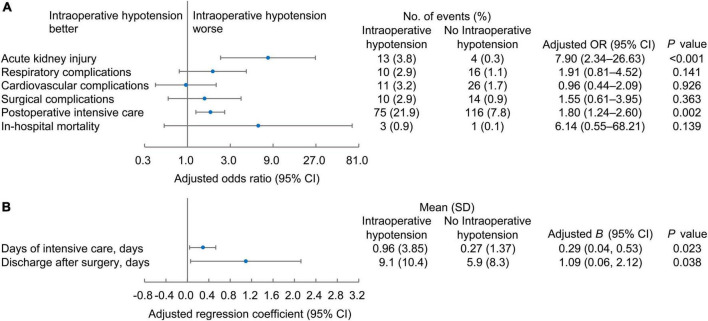
The binary **(A)** and continuous **(B)** postoperative outcomes of patients according to the occurrence of intraoperative hypotension. Forest plots of the adjusted odd ratios or beta coefficient **(B)** values with 95% confidence intervals.

After adjusting for the selected confounders (age, male, body mass index, ASA-PS classification, number of spine levels treated, blood loss, hypertension, and diabetes), the presence of intraoperative hypotension significantly influenced postoperative adverse outcomes, including more acute kidney injury (OR, 7.90; 95% CI, 2.34–26.63, *P* < 0.001), more postoperative critical care need (OR, 1.80; 95% CI, 1.24–2.60, *P* = 0.002) (Figure 3A), longer ICU stay (0.29 days longer, 95% CI 0.04–0.53, *P* = 0.023), and longer interval between surgery and discharge (1.09 days longer, 95% CI 0.06-2.12, *P* = 0.038) (Figure 3B).

## Discussion

In adult patients who underwent non-emergency decompression surgery for cervical spondylosis, intraoperative hypotension and vasopressor requirement were common (43%), and severe hypotension occurred in 18.7% of patients. We showed that a history of chronic arterial hypertension was associated with both post-induction and post-incision hypotension. Surgical features, including the treatment of the upper cervical spine (C0-2) and increased number of spine segments, were predictive of both post-induction and post-incision hypotension. Patients who developed intraoperative hypotension experienced more acute postoperative kidney injury, greater need for intensive care, and longer admission after surgery.

Chronic arterial hypertension was defined as medical history in this study. The prevalence of chronic arterial hypertension was approximately 43% in our study, slightly higher than the prevalence reported by Strapelfeldt et al. (40%) in general surgical patients (8), but lower than the reported incidence of approximately two-thirds in another study that used in-hospital ambulatory blood pressure, rather than medical history, to define hypertension based on recommendations by the Hypertension Society (7). The use of historical data may underestimate the prevalence of hypertension compared to monitoring ambulatory blood pressure for diagnosis. However, use of in-hospital ward blood pressure measurement as the diagnosis can also overestimate the prevalence of hypertension. Thus, the definition of hypertension in our study were appropriate for identification of the risk factors of intraoperative hypotension.

Our results are consistent with the findings of previous studies showing that chronic hypertension predicts intraoperative hypotension in the general surgical population (7, 8). Intraoperative hypotension can be caused by numerous factors, including vasodilation or low cardiac output (due to anesthetic drugs and systematic infection), high intrathoracic pressure, impairment of the sympathetic nervous system, or compromised baroreflex regulation (23). Hypertension frequently co-exists with comorbidities such as chronic kidney disease, diabetes, and hypercholesterolemia (26, 27), and hypertensive patients are more vulnerable to dysfunctional autonomic cardiovascular control (9) and asymptomatic cerebrovascular damage (28), leading to intraoperative hypotension. One study that aimed to characterize hazardous intraoperative hypotension demonstrated that patients with a preoperative diagnosis of hypertension required less cumulative intraoperative hypotension time to incur the same increase in 30-day mortality compared to patients without a history of hypertension (8). Thus, hypertensive patients in advanced stages are susceptible to hypotension due to inadequate physiologic compensation to intraoperative events such as anesthetics. The sympathetic nervous system fails to properly adjust the tone in the arteries, veins, and heart; consequently, intraoperative hypotension develops and persists, requiring vasopressor treatment.

Our results were consistent with previously reported predictors for intraoperative hypotension other than chronic hypertension, including age, male sex, and history of congestive heart failure (4). Our results also revealed surgery-related features predicting hypotension, such as an upper cervical spine level (C0-2) and an increased number of segments treated. It is not known whether cervical spondylosis at the upper cervical level is associated with the development of hypertension or autonomic dysfunction more than that involving the lower cervical spine level. An animal study revealed that atlantoaxial misalignment could cause high blood pressure (29) and a case report described neurogenic hypertension related to brain stem invagination by the upper cervical spine (30); thus, development of clinical hypertension or autonomic neuropathy after long-term cord compression of the upper cervical cord is possible. Whether these patients are more vulnerable to intraoperative hypotension because of autonomic dysfunction remains unclear and requires further investigation.

In this study, we used an absolute threshold value of SAP < 80 mmHg for low SBP and defined severe hypotension as two consecutive readings of low SBP and more than two incidents of vasopressor treatment. A few studies support this approach, showing that definitions of hypotension using absolute ABP thresholds lead to similar conclusions when assessing a patient’s risk of intraoperative hypotension or related end-organ damage, in comparison with a relative decline from an averaged preoperative ABP baseline value (4, 31). Considering previous investigations on the association between cervical spondylotic myelopathy and chronic hypertension, particularly systolic arterial hypertension, the definition seemed to be pathophysiologically sound and pragmatic with respect to our study design for risk assessment.

The postoperative outcomes revealed an increased incidence of acute kidney injury in patients with intraoperative hypotension; however, there were no differences in cardiovascular complications and in-hospital mortality. This may be attributed to the fact that a considerable proportion of our patients (79%) underwent arterial catheter pressure monitoring. Hypotension can be detected earlier in patients with arterial catheter pressure monitoring, and these patients can receive more vasopressor boluses than those with oscillometric pressure monitoring (3). Our results helped estimate the risks for vulnerability to severe and persistent hypotension, and early treatments should be initiated to prevent adverse outcomes.

Our study had several limitations. Our data were collected from patients at a single university medical center and treated according to local clinical standards. Therefore, our results lack external validation. For example, ephedrine and norepinephrine are vasopressors of choice for the stabilization of anesthesia-related blood pressure instability in our department, whereas different agents might be used to this end in other institutions. In addition, other unique local practices concerning approaches for individual patients may be implicit in nature and thus not measurable, although they may influence the occurrence and independent variables of post-induction and post- incision hypotension. Furthermore, anesthetic management was not standardized in this retrospective study. Additionally, to avoid overfitting the multivariable regression model, we included only a limited number of independent variables that we thought were clinically meaningful (32). Thus, it is likely that we missed at least some potential confounding characteristics. As with any prediction model, our findings must be confirmed and externally validated in other independent patient cohorts.

## Conclusion

In conclusion, we characterized intraoperative arterial hypotension in patients who underwent non-emergency decompression surgery for cervical spondylosis. We demonstrated that older age, male sex, history of hypertension, and surgical features such as upper cervical spine levels and increased number of segments treated were factors independently associated with both post-induction and post- incision hypotension. Patients diagnosed with OPLL were more likely to have a history of hypertension. These findings may enable pre-emptive risk optimization through the early implementation of continuous hemodynamic monitoring and interventions.

## Implication for practice and research

Intraoperative hypotension is associated with negative postoperative outcomes, and an understanding of the risk factors for intraoperative hypotension can facilitate early detection and timely treatment to minimize the hypotensive period. However, previous predictions of intraoperative hypotension did not focus on a specific surgical population. Previously a cause effect relationship between cervical spondylotic myelopathy and chronic hypertension was postulated and thus we speculate whether patients with chronic hypertension history are more susceptible to hypotension after anesthetic induction. Our study makes a significant contribution because the results well characterized the risk factors for intraoperative hypotension pertaining to surgical features. Prompt assessments for identifiable features can help ameliorate intraoperative hypotension.

## Data availability statement

The original contributions presented in this study are included in the article/Supplementary material, further inquiries can be directed to the corresponding author.

## Author contributions

T-YC helped design the study and collect the data and draft the manuscript. Y-KW helped collect the data and draft the manuscript. W-CH helped analyze and interpret the data and draft the manuscript. S-SH helped design the study and analyze and interpret the data and revise the manuscript. Y-CC helped design the study, collect the data, analyzed and interpret the data and finalize the manuscript. All authors edited the draft, revised, and approved the manuscript.

## References

[1] Monk TG Bronsert MR Henderson WG Mangione MP Sum-Ping ST Bentt DR Association between intraoperative hypotension and hypertension and 30-day postoperative mortality in noncardiac surgery. *Anesthesiology.* (2015) 123:307–19. 10.1097/ALN.0000000000000756 26083768

[2] Walsh M Devereaux PJ Garg AX Kurz A Turan A Rodseth RN Relationship between intraoperative mean arterial pressure and clinical outcomes after noncardiac surgery: toward an empirical definition of hypotension. *Anesthesiology.* (2013) 119:507–15. 10.1097/ALN.0b013e3182a10e26 23835589

[3] Naylor AJ Sessler DI Maheshwari K Khanna AK Yang D Mascha EJ Arterial catheters for early detection and treatment of hypotension during major noncardiac surgery: a randomized trial. *Anesth Analg.* (2020) 131:1540–50. 10.1213/ANE.0000000000004370 33079877

[4] Südfeld S Brechnitz S Wagner JY Reese PC Pinnschmidt HO Reuter DA Post-induction hypotension and early intraoperative hypotension associated with general anaesthesia. *Br J Anaesth.* (2017) 119:57–64. 10.1093/bja/aex127 28974066

[5] Reich DL Hossain S Krol M Baez B Patel P Bernstein A Predictors of hypotension after induction of general anesthesia. *Anesth Analg.* (2005) 101:622–8. 10.1213/01.ANE.0000175214.38450.91 16115962

[6] Taffé P Sicard N Pittet V Pichard S Burnand B, Ads study group. The occurrence of intra-operative hypotension varies between hospitals: observational analysis of more than 147,000 anaesthesia. *Acta Anaesthesiol Scand.* (2009) 53:995–1005. 10.1111/j.1399-6576.2009.02032.x 19572938

[7] Hoppe P Burfeindt C Reese PC Briesenick L Flick M Kouz K Chronic arterial hypertension and nocturnal non-dipping predict postinduction and intraoperative hypotension: a secondary analysis of a prospective study. *J Clin Anesth.* (2022) 79:110715. 10.1016/j.jclinane.2022.110715 35306353

[8] Stapelfeldt WH Yuan H Dryden JK Strehl KE Cywinski JB Ehrenfeld JM The SLUScore: a novel method for detecting hazardous hypotension in adult patients undergoing noncardiac surgical procedures. *Anesth Analg.* (2017) 124:1135–52. 10.1213/ANE.0000000000001797 28107274 PMC5367493

[9] Mancia G Grassi G. The autonomic nervous system and hypertension. *Circ Res.* (2014) 114:1804–14. 10.1161/CIRCRESAHA.114.302524 24855203

[10] Frandsen MN Mehlsen J Bang Foss N Kehlet H. Pre-operative autonomic nervous system function - a missing link for post-induction hypotension? *Anaesthesia.* (2022) 77:139–42. 10.1111/anae.15546 34291821

[11] Li ZQ Zhao YP Jia WY Wang X Chen B Shahbaz M Surgical treatment of cervical spondylotic myelopathy associated hypertension-a retrospective study of 309 patients. *PLoS One.* (2015) 10:e0133828. 10.1371/journal.pone.0133828 26193469 PMC4508105

[12] Yang L Yang C Pang X Li D Chen X Shi J Cervical decompression surgery for cervical spondylotic myelopathy and concomitant hypertension: a multicenter prospective cohort study. *Spine.* (2017) 42:903–8. 10.1097/BRS.0000000000001941 27792119

[13] Itoki K Kurokawa R Shingo T Kim P. Effect of myoarchitectonic spinolaminoplasty on concurrent hypertension in patients with cervical spondylotic myelopathy. *Neurospine.* (2018) 15:77–85. 10.14245/ns.1836020.010 29656621 PMC5944632

[14] Li P Wei Z Zhang H Zhang K Li J. Effects of decompressive operation on cardiac autonomic regulation in patients with cervical spondylotic myelopathy: analysis of blood pressure, heart rate, and heart rate variability. *Eur Spine J.* (2019) 28:1864–71. 10.1007/s00586-019-05972-9 31011802

[15] Kalb S Zaidi HA Ribas-Nijkerk JC Sindhwani MK Clark JC Martirosyan NL Persistent outpatient hypertension is independently associated with spinal cord dysfunction and imaging characteristics of spinal cord damage among patients with cervical spondylosis. *World Neurosurg.* (2015) 84:351–7. 10.1016/j.wneu.2015.03.030 25819526

[16] Lin SY Hsu WH Lin CC Lin CL Tsai CH Lin CH Association of arrhythmia in patients with cervical spondylosis: a nationwide population-based cohort study. *J Clin Med.* (2018) 7:236. 10.3390/jcm7090236 30142924 PMC6162845

[17] Lin SY Chen DC Lin CL Lee HC Lin TC Wang IK Risk of acute coronary syndrome in patients with cervical spondylosis. *Atherosclerosis.* (2018) 271:136–41. 10.1016/j.atherosclerosis.2018.02.029 29518745

[18] Lin WS Huang TF Chuang TY Lin CL Kao CH. Association between cervical spondylosis and migraine: a nationwide retrospective cohort study. *Int. J. Environ Res Public Health.* (2018) 15:587. 10.3390/ijerph15040587 29587400 PMC5923629

[19] Srihari G Shukla D Indira Devi B Sathyaprabha TN. Subclinical autonomic nervous system dysfunction in compressive cervical myelopathy. *Spine (Phila Pa 1976).* (2011) 36:654–9. 10.1097/BRS.0b013e3181dc9eb2 21178837

[20] Yamada H Honda T Yaginuma H Kikuchi S Sugiura Y. Comparison of sensory and sympathetic innervation of the dura mater and posterior longitudinal ligament in the cervical spine after removal of the stellate ganglion. *J Comp Neurol.* (2001) 434:86–100. 10.1002/cne.1166 11329131

[21] Li J Gu T Yang H Liang L Jiang DJ Wang ZC Sympathetic nerve innervation in cervical posterior longitudinal ligament as a potential causative factor in cervical spondylosis with sympathetic symptoms and preliminary evidence. *Med Hypotheses.* (2014) 82:631–5. 10.1016/j.mehy.2014.02.029 24629355

[22] Johnson GM. The sensory and sympathetic nerve supply within the cervical spine: review of recent observations. *Man Ther.* (2004) 9:71–6. 10.1016/S1356-689X(03)00093-6 15040965

[23] Bijker JB van Klei WA Kappen TH van Wolfswinkel L Moons KG Kalkman CJ. Incidence of intraoperative hypotension as a function of the chosen definition - Literature definitions applied to a retrospective cohort using automated data collection. *Anesthesiology.* (2007) 107:213–20. 10.1097/01.anes.0000270724.40897.8e 17667564

[24] Kouz K Hoppe P Briesenick L Saugel B. Intraoperative hypotension: pathophysiology, clinical relevance, and therapeutic approaches. *Indian J Anaesth.* (2020) 64:90–6. 10.4103/ija.IJA_939_19 32139925 PMC7017666

[25] Khwaja A. KDIGO clinical practice guidelines for acute kidney injury. *Nephron Clin Pract.* (2012) 120:C179–84. 10.1159/000339789 22890468

[26] Wong ND Lopez VA L’Italien G Chen R Kline SE Franklin SS. Inadequate control of hypertension in US adults with cardiovascular disease comorbidities in 2003-2004. *Arch Intern Med.* (2007) 167:2431–6. 10.1001/archinte.167.22.2431 18071164

[27] Guthrie B Payne K Alderson P McMurdo ME Mercer SW. Adapting clinical guidelines to take account of multimorbidity. *Br Med J.* (2012) 345:e6341. 10.1136/bmj.e6341 23036829

[28] Kohara K Jiang Y Igase M Takata Y Fukuoka T Okura T Postprandial hypotension is associated with asymptomatic cerebrovascular damage in essential hypertensive patients. *Hypertension.* (1999) 33:565–8. 10.1161/01.HYP.33.1.565 9931166

[29] He ZB Lv YK Li H Yao Q Wang KM Song XG Atlantoaxial misalignment causes high blood pressure in rats: a novel hypertension model. *Biomed Res Int.* (2017) 2017:5986957. 10.1155/2017/5986957 28791307 PMC5534292

[30] Dickinson LD Papadopoulos SM Hoff JT. Neurogenic hypertension related to basilar impression. Case report. *J Neurosurg.* (1993) 79:924–8. 10.3171/jns.1993.79.6.0924 8246061

[31] Salmasi V Maheshwari K Yang D Mascha EJ Singh A Sessler DI Relationship between intraoperative hypotension, defined by either reduction from baseline or absolute thresholds, and acute kidney and myocardial injury after noncardiac surgery: a retrospective cohort analysis. *Anesthesiology.* (2017) 126:47–65. 10.1097/ALN.0000000000001432 27792044

[32] Riley RD Ensor J Snell KIE Harrell FE Jr. Martin GP Reitsma JB Calculating the sample size required for developing a clinical prediction model. *Br Med J.* (2020) 368:m441. 10.1136/bmj.m441 32188600

