# Supplementary material for: Intraoperative hypotension in non-emergency decompression surgery for cervical spondylosis: The role of chronic arterial hypertension
Source: Front Med (Lausanne). 2022 Oct 18;9:943596. doi: 10.3389/fmed.2022.943596 (PMC9622940; doi:10.3389/fmed.2022.943596)
Supplement: Supplementary file 1 [file Table_1.DOCX]

**Supplemental Table S1**. Factors associated with post-induction and post-incision hypotension using univariate logistic regression

|  | Post-induction hypotension | | |  | Post-incision hypotension | | |
| --- | --- | --- | --- | --- | --- | --- | --- |
| Variable | Crude OR (95% CI) | | *P* |  | Crude OR (95% CI) | | *P* |
| Age, years | 1.04 | (1.03–1.06) | <0.001 |  | 1.05 | (1.04–1.06) | <0.001 |
| Male | 1.67 | (1.26–2.21) | <0.001 |  | 1.72 | (1.31–2.26) | <0.001 |
| Body mass index, kg/m^2^ | 1.03 | (1.00–1.06) | 0.095 |  | 1.02 | (0.99–1.06) | 0.134 |
| ASA (3&4 vs. 1&2) | 1.74 | (1.29–2.36) | <0.001 |  | 2.04 | (1.53–2.72) | <0.001 |
| Clinical myelopathy | 1.31 | (1.004–1.71) | 0.047 |  | 1.32 | (1.02–1.71) | 0.034 |
| Cervical level treated (C0-C2 vs. C3-T1) | 3.62 | (2.37–5.52) | <0.001 |  | 2.94 | (1.92–4.50) | <0.001 |
| Number of spine segments treated | 1.63 | (1.44–1.84) | <0.001 |  | 1.61 | (1.43–1.82) | <0.001 |
| Current smoker | 1.08 | (0.79–1.47) | 0.636 |  | 1.02 | (0.75–1.38) | 0.916 |
| Pre-induction SBP, per 100 mmHg | 1.64 | (0.87–3.09) | 0.126 |  | 1.72 | (0.93–3.17) | 0.082 |
| Propofol, mg/kg | 0.62 | (0.47–0.82) | <0.001 |  | – |  | – |
| Blood loss, per 100 ml | – |  | – |  | 1.21 | (1.16–1.27) | <0.001 |
| Hypertension | 2.39 | (1.82–3.14) | <0.001 |  | 2.42 | (1.86–3.14) | <0.001 |
| OPLL | 1.82 | (1.29–2.58) | <0.001 |  | 1.99 | (1.42–2.77) | <0.001 |
| Diabetes | 1.39 | (1.02–1.90) | 0.040 |  | 1.41 | (1.04–1.90) | 0.026 |
| Chronic kidney disease | 1.98 | (0.96–4.08) | 0.064 |  | 1.78 | (0.87–3.67) | 0.116 |
| Congestive heart failure | 4.19 | (1.18–14.97) | 0.027 |  | 1.41 | (0.30–6.66) | 0.667 |
| Coronary heart disease | 1.29 | (0.84–1.99) | 0.240 |  | 1.45 | (0.97–2.17) | 0.073 |
| Vasodilator | 1.12 | (0.55–2.31) | 0.752 |  | 1.79 | (0.97–3.30) | 0.064 |
| CCB | 1.13 | (0.83–1.55) | 0.441 |  | 1.18 | (0.87–1.60) | 0.283 |
| ACEi / ARB | 1.08 | (0.37–3.16) | 0.885 |  | 1.07 | (0.79–1.45) | 0.651 |
| Diuretic/ Thiazide | 1.52 | (0.86–2.66) | 0.146 |  | 1.15 | (0.64–2.07) | 0.648 |
| Beta blocker | 0.59 | (0.36–0.97) | 0.036 |  | 0.85 | (0.56–1.30) | 0.463 |
| Alpha blocker | 1.07 | (0.41–2.80) | 0.885 |  | 0.75 | (0.26–2.13) | 0.584 |

Abbreviations: OR, odds ratio; CI, confidence interval; ASA, American Society of Anesthesiologists; SBP, systolic blood pressure; OPLL, ossification of the posterior longitudinal ligament; CCB, calcium-channel blocker; ACEi, angiotensin-converting enzyme inhibitor; ARB, angiotensin receptor blocker.
